# Testing implementation support for evidence-based programs in community settings: a replication cluster-randomized trial of Getting To Outcomes®

**DOI:** 10.1186/s13012-018-0825-7

**Published:** 2018-10-22

**Authors:** Matthew Chinman, Patricia Ebener, Patrick S. Malone, Jill Cannon, Elizabeth J. D’Amico, Joie Acosta

**Affiliations:** RAND Corporation, 4570 Fifth Avenue, Pittsburgh, PA 15213 USA

**Keywords:** Implementation support, Fidelity, Evidence-based prevention, Community-based

## Abstract

**Background:**

Community organizations can have difficulty implementing evidence-based prevention programs. More research is needed on implementation support interventions designed to help these organizations implement programs with quality.

**Methods:**

Preparing to Run Effective Programs (PREP) is a randomized controlled trial testing Getting To Outcomes (GTO), a 2-year implementation support intervention. It compares 15 Boys and Girls Club sites implementing CHOICE (control group), a five-session evidence-based alcohol and drug prevention program, with 14 similar sites implementing CHOICE supported by GTO (intervention group). PREP replicates a previous GTO study that had the same design, but featured a teen pregnancy prevention program instead. All sites received typical CHOICE training. Fourteen intervention sites received GTO manuals, training, and onsite technical assistance to help practitioners complete implementation best practices specified by GTO (i.e., GTO steps). During the first year, technical assistance providers helped the intervention group adopt, plan, and deliver CHOICE. Then, this group was trained on evaluation and quality improvement steps of GTO using feedback reports summarizing their own data, which yielded revised plans for subsequent implementation of CHOICE. This paper presents results regarding GTO’s impact on CHOICE fidelity (adherence, quality of delivery, dosage) and the proximal outcomes of the youth participants (aged 10–14)—attitudes and intentions regarding cigarettes, alcohol, and marijuana use. Fidelity was assessed at all sites by observer ratings and attendance logs. Proximal outcomes were assessed via survey at baseline, 3, and 6 months.

**Results:**

After 1 year, fidelity and proximal outcomes were similar between Intervention and control groups. After 2 years (which included GTO quality improvement activities that took place between years 1 and 2), intervention sites had higher ratings of CHOICE adherence and quality of delivery (dosage remained similar). Proximal outcomes did not differ between groups in either year, although there was universally high endorsement of prosocial responses to those outcomes from the start.

**Conclusions:**

Findings suggest that systematic implementation support provided by GTO can help community organizations achieve better fidelity. Findings replicate the implementation results from a previous GTO study using the same design, but with a different evidence-based program and different fidelity measures. Although proximal outcomes did not change, in large part due to ceiling effects, the implementation findings suggest GTO can support a variety of programs.

**Trial registration:**

This project is registered at ClinicalTrials.gov with number NCT02135991. The trial was first registered on May 12, 2014.

Problematic rates of alcohol, marijuana, and other drug use among US adolescents highlight the need for good implementation of prevention evidence-based programs. In 2015, over half of high school seniors reported alcohol use in the past year, with one third reporting being drunk in this time frame. One third of high school seniors report past month drinking, and over 20% report using marijuana monthly [1]. Also, the use of opioids has reached epidemic proportions, and electronic cigarette use has skyrocketed in the past 2 years, outpacing the use of regular cigarettes among youth [1]. The estimated costs of alcohol misuse, illicit drug use, and substance use disorders are more than $400 billion [2]. Despite the need and availability of scores of alcohol and drug prevention evidence-based programs (see the Penn State Clearinghouse, https://militaryfamilies.psu.edu/programs-review/), communities often face difficulty implementing evidence-based programs with the quality needed to achieve outcomes [3–9]. This poor implementation often results from limited resources and a lack of capacity—the knowledge, attitudes, and skills—individual practitioners need to implement “off the shelf” evidence-based programs.

Strong implementation includes best practices, such as setting realistic goals, thoughtful planning, evaluation, quality improvement, and program sustainability. Many youth-serving organizations require help with these practices. Preparing to Run Effective Prevention (PREP) is a 2-year, randomized controlled trial of an implementation support intervention called Getting To Outcomes® or GTO [10], which is designed to build capacity for these practices. The aim of the PREP study was to test GTO’s impact on fidelity and youth outcomes of an evidence-based, substance use prevention program called CHOICE [11], carried out by community-based, youth-serving organizations. PREP is a replication of an earlier GTO study, Enhancing Quality Interventions Promoting Healthy Sexuality (EQUIPS, [12]) in which the evidence-based program was a teen pregnancy prevention program called Making Proud Choices [13].

## Getting to outcomes—an implementation support intervention

**Fig. 1:**
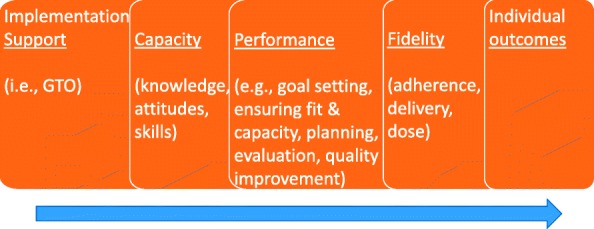
Getting To Outcomes logic model

GTO builds capacity for implementing evidence-based programs by strengthening the knowledge, attitudes, and skills needed to carry out implementation best practices for running any program [14]—i.e., goal setting, planning, evaluation, quality improvement, and sustaining. Rooted in social cognitive theories of behavioral change [15–18] and implementation science theories such as the Consolidated Framework for Implementation Research (see [19, 20]), GTO’s logic model (see Fig. 1) states that GTO training and technical assistance builds practitioner capacity to perform multiple implementation best practices needed for an evidence-based program (see Table 1) [21]. Improved performance of these implementation best practices when delivering a specific evidence-based program can improve program fidelity, which results in more positive outcomes [21].

**Table 1:** Manual information and practices performed by BGC staff by each of the 10 GTO steps

GTO step	What the GTO manual provides for each step	Practices BGC club staff carried out within each GTO step
1. Needs: what are the needs to address and the resources that can be used?	Information about how to conduct a needs and resources assessment	Club staff reviewed data about the needs of their membership
2. Goals and outcomes: what are the goals and desired outcomes?	Tools for creating measurable goals and desired outcomes	Each site developed their own broad goals and “desired outcomes”—statements that specify the amount and timing of change expected on specific measures of knowledge, attitudes, behavior
3. Best practices: which evidence-based programs can be useful in reaching the goals?	Overview of the importance of using evidence-based programs and where to access information about them	Club leaders agreed to use CHOICE as the evidence-based program to implement
4. Fit: what actions need to be taken so the selected program fits the community context?	Tools to help program staff identify opportunities to reduce duplication and facilitate collaboration with other programs.	Each site reviewed CHOICE for how it would fit within their club and made adaptations to improve fit
5. Capacity: what capacity is needed for the program?	Assessment tools to help program staff ensure there is sufficient organizational, human and fiscal capacity to conduct the program	Each site assessed their own capacity to carry out CHOICE and made plans to increase capacity when needed
6. Plan: what is the plan for this program?	Information and tools to plan program activities in detail	Each site conducted concrete planning for doing CHOICE (e.g., who, what, where, when)
7. Process evaluation: how will the program implementation be assessed?	Information and tools to help program staff plan and implement a process evaluation	Each site collected data on fidelity, attendance, satisfaction to assess program delivery and reviewed that data immediately after implementation
8. Outcome evaluation: how well did the program work?	Information and tools to help program staff implement an outcome evaluation	Each site collected participant outcome data on actual behavior as well as on mediators such as attitudes and intentions
9. Continuous quality improvement: how will continuous quality improvement strategies be used to improve the program?	Tools to prompt program staff to reassess GTO steps 1–8 to stimulate program improvement plans	Each site reviewed decisions made and tools completed before implementation and data collected during and after implementation and made concrete changes for the next implementation
10. Sustainability: if the program is successful, how will it be sustained?	Ideas to use when attempting to sustain an effective program	Each site considered such as securing adequate funding, staffing, and buy-in, to make it more likely that CHOICE would be sustained

GTO was developed by Chinman, Imm, and Wandersman as a written guide and published by the RAND Corporation in 2004 to help individuals conduct drug and alcohol prevention programs [22], https://www.rand.org/pubs/technical_reports/TR101.html. It was developed by reviewing multiple literatures on planning, implementation, and program evaluation and then distilling down key points that could be more easily understood by community-based practitioners [23]. Also, tools—or worksheets—were added to the guide to prompt users to make and record key decisions. As part of the first GTO study, a quasi-experimental trial from 2002 to 2005 [24], RAND added face to face training and ongoing technical assistance to the existing written guide to increase GTO’s impact. From then on, in all subsequent studies, the GTO approach provides three supports: (1) the GTO manual (tailored to a variety of content domains including drug and alcohol prevention, which was used in PREP, [22]), (2) face-to-face training, and (3) ongoing, onsite, and proactive technical assistance. GTO has been applied to multiple content areas including teen pregnancy prevention [25], underage drinking prevention [26], and positive youth development [27].

Key to GTO’s capacity-building is asking practitioners to be active learners. GTO establishes expectations and gives opportunities and guidance for practitioners to carry out for themselves the implementation best practices that GTO specifies.

In previous quasi-experimental [24] and randomized controlled trials [19, 28], GTO has been found to improve capacity of individual practitioners and performance of alcohol and drug prevention programs. However, those studies involved mostly non-evidence-based programs of widely varying type and quality, and thus were not able to assess common outcomes across program participants. The EQUIPS study had the same design as PREP and showed that community-based organizations (Boys and Girls Clubs) using GTO demonstrated better capacity, performance, fidelity, and youth outcomes from a teen pregnancy prevention evidence-based program (Making Proud Choices) than clubs not using GTO [12, 29]. Other work has also demonstrated that implementation support can improve fidelity and outcomes of substance use prevention evidence-based programs, but those trials were not able to track programming being implemented, or its fidelity, in the control communities [30, 31] or track technical assistance usage and blind fidelity observers [32].

## Contributions of the PREP study

PREP builds upon past studies of implementation support in general, and of GTO in particular. The PREP design replicates the EQUIPS study (cluster randomized controlled trial comparing evidence-based program vs evidence-based program+GTO), but does so using a different evidence-based program (i.e., CHOICE) in a different content domain (i.e., substance use), with some similar and some different measures of fidelity and outcomes (access to the CHOICE developer, ED, was helpful to ensure we used the same measures as in past CHOICE trials). Replicating findings in a different content domain represents a strong test of GTO’s robustness. In particular, testing the fidelity of CHOICE included examining fidelity to motivational interviewing, a non-judgmental, non-confrontational counseling approach typically used with a variety of health risk behaviors [33, 34]. Given that CHOICE program delivery utilizes motivational interviewing [35], delivering the program involves a more complicated set of skills than many universal prevention programs require, and thus presents a greater challenge to achieve program fidelity. Finally, PREP, like EQUIPS, has rigorous design features that past GTO and other implementation support studies have not been able to incorporate including use of a single evidence-based program to reduce variation between intervention and control groups, measures of implementation (fidelity) in the intervention *and* control groups, fidelity observers blinded to group condition, and measures of both fidelity and individual outcomes [19, 24, 28, 36–39].

## Methods

### Design overview

PREP is a 2-year randomized controlled trial (RCT) comparing 15 sites within 8 Boys and Girls Clubs (BGCs) who received typical training to implement the CHOICE program [11] (control group) with 14 sites within seven BGCs who received the same CHOICE training, plus GTO manuals, training, and technical assistance (intervention group). As in EQUIPS, GTO was provided over a 2-year period, allowing all sites to deliver CHOICE twice. The trial assessed fidelity (e.g., adherence, quality of CHOICE delivery, dosage) and the alcohol and drug outcomes of participating middle school youth. Based on results from the EQUIPS trial [40, 41], it was hypothesized that the intervention sites would be higher on fidelity than the control sites in the second year, and that the youth in the intervention sites would show more improvement in alcohol and drug outcomes than youth in control sites in the second year.

### Study sites

The 29 sites are in the greater Los Angeles, California area, covering Los Angeles (23 sites) and Orange (six sites) counties. BGCs provide youth programming ranging from recreation in gyms to leadership, character education, health and wellness, and academic programs. A BGC often has several sites (i.e., geographic locations). Despite some variability, each site typically has its own facility and a small number of full- and part-time staff (*n* = 7–10). A sub-set of staff (between 1 and 10; mean = 2.2, median = 3) at each site participated in the study. CHOICE was initially developed in Los Angeles with a diverse population of youth [35, 42] and was therefore appropriate for the mostly Latino and African-American sample found in these BGC sites. Invitations were made to all BGCs in the area (*n* = 38) via meetings of a BGC alliance. The study team stopped recruiting when the above sample was reached. The site level sample size was justified at 80% power by taking into account the estimated correlation between baseline and follow-up assessments of the site level measures (.5 to .6) and the moderate to large effect sizes expected based on previous GTO [40] and CHOICE [11] studies.

### Youth sample

The youth level sample size was justified using data from previous CHOICE trials [11], including the expected correlation between baseline and follow-up assessments (*r* = 0.4), the intraclass correlation measure of clustering (ICC = 0.3), and the small to medium effect sizes achieved on various outcome measures of interest.

In study year 1, 356 youth in self-reported grades 7–9 participated in the youth survey. These youth ranged in age from 10 to 14 (*M* = 11.9, SD = 1.0); 48% were in grade 7, 37% in grade 8, and 15% in grade 9. Gender was 50% girls. The survey requested binary responses to ethnicity (Hispanic or Latino/Latina vs. not) and six separate racial identifications; youth were permitted to choose all that applied. Sixty-four percent reported Latinx ethnicity; 17% reported being Black or African American; 13% White or Caucasian; 9% Asian or Asian American; 6% American Indian or Alaska Native, and less than 5% Native Hawaiian or Pacific Islander. “Other race” was selected by 58% of respondents; 86% of these youth indicated Hispanic or Latinx ethnicity. Multiple racial identifications were indicated by 7% of youth.

In year 2, *n* = 253, ranging in age from 10 to 15 (*M* = 11.9, SD = 1.0); 55% were in grade 7, 38% in grade 8, and 8% in grade 9. Approximately half (51%) were girls. Sixty-six percent reported Latinx ethnicity; 14% were Black or African American; 12% Asian or Asian American; 11% White or Caucasian; 8% American Indian or Alaska Native, and less than 5% Native Hawaiian or Pacific Islander. “Other race” was selected by 59%; 90% of these youth were Hispanic or Latinx. Multiple races were selected by 7% of youth.

### Procedures

Using a random number generator, we randomized the 15 BGC clubs to intervention (8 BGCs—15 sites) or control (7 BGCs—14 sites). We randomized at the BGC level (versus site) to minimize potential contamination due to communication between sites within the same club system. The principal investigator informed each club about their assignment.

At baseline (after randomization), we conducted a web-based survey of BGC staff involved in CHOICE to assess for potential differences in demographic variables and attitudes toward evidence-based programs. All staff who were contacted, responded (control = 29/29; intervention = 34/34). Staff in the control and intervention groups had largely similar demographic makeup (no significant differences based on bivariate models accounting for clustering within BGC and county). Half (49%) of the staff were female; most (59%) were over 25 years old; half (50%) had a 4-year college degree or more; and 56% were Hispanic or Latinx, 22% were non-Hispanic African-American and 22% were non-Hispanic White, multiracial, or of other races. Over a third (38%) were full-time employees at their respective BGC site.

The web survey included the Evidence-Based Practice Attitude Scale [43]. Its four scales, and their McDonald’s ω coefficients in this study (with 95% confidence intervals [CIs]), assess the degree to which practitioners would adopt an evidence-based program based on the following factors: appeal (how much the evidence-based program was intuitively attractive), ω = .65, CI [.47, .83]; requirements (degree to which it was considered mandatory), ω = .88 [.7, .94]; openness (willingness to trying new interventions), ω = .77 [.66, .85]; and divergence (belief that experience is more important than research), ω = .81 [.66, .89]). Coefficient ω [44] is a measure of internal consistency on the same scale as coefficient alpha, but makes less biased estimations, has fewer problems with inflation due to number of items, and has CIs to more accurately evaluate reliability [45]. The ω values found here are considered acceptable to good [46]. To evaluate baseline group differences on each scale, we fit a linear mixed-effects regression model with fixed treatment effect (intervention vs. control) and a random club (BGC) and county (Los Angeles vs. Orange) intercepts. There were no significant differences between the two groups on any scale at baseline, *p*s > .2, with or without staff-level demographic covariates.

### CHOICE—an evidence-based alcohol and drug prevention program

CHOICE involves five, half-hour sessions based on social learning theory [47], decision-making theory [48], and self-efficacy theory [49]. Delivered using motivational interviewing [50], CHOICE uses role plays to teach resistance skills and discuss pros and cons of cutting down and stopping use. The five sessions cover (1) normative feedback on alcohol and drug use among middle-school youth; (2) how beliefs about substances can affect behavior and how substances affect people; (3) how substances can be used to deal with stress or other negative emotions and how social substance use can become problematic; (4) how to identify certain pressures to use substances and how to resist them; and (5) how to use problem solving skills to avoid using substances when they are present [35]. CHOICE training consists of two, mostly full-day sessions on content and motivational interviewing role playing. The evidence for CHOICE comes from two randomized trials that found the program was associated with reductions in alcohol and marijuana use [11, 51].

### CHOICE implementation supported by GTO

Using existing staff, each BGC site was asked to implement CHOICE once a year for 2 years with a different group of adolescents each year between May 2014 and April 2016. Two half-time, masters level technical assistance providers delivered standard CHOICE manuals and training to all sites. For intervention sites, technical assistance providers also delivered GTO manuals, face-to-face training in GTO, and onsite technical assistance with phone and email follow-up to support implementation during the two rounds of CHOICE delivery (per site in year 1: *M* = 11.17 h of TA, SD = 3.4; year 2: *M* = 14.7 h of TA, SD = 3.9). The GTO manual contains written guidance about how to complete multiple implementation best practices important for evidence-based programs—i.e., GTO steps. Most GTO steps contain tools that prompt practitioners to make, and then record, decisions about various practices. For example, the GTO Goals Tool has prompts that assist practitioners to write goal and desired outcome statements. Table 1 shows how BGC staff assigned to the GTO condition performed the various implementation practices in each of the GTO steps to implement CHOICE.

Before the first CHOICE implementation, technical assistance providers delivered two GTO trainings to participating staff at each intervention site. The first focused on GTO steps 1–3 (needs assessment, setting goals and concrete objectives, and best practices). A few weeks later, each site received training on GTO steps 4–6, focusing on program fit, capacity, and planning. Concurrently, technical assistance providers met periodically with BGC staff to help them complete each GTO step (i.e., complete the tools) and guide the planning of CHOICE. Then, BGC staff at all sites implemented CHOICE and facilitated the collection of fidelity and youth outcome data (described below). Intervention sites then received training on evaluation and quality improvement (GTO steps 7–9), along with feedback reports summarizing fidelity and youth outcome data from their sites, which were used in a TA-facilitated quality improvement process that resulted in a revised plan for the second implementation of CHOICE. The year 2 implementation followed the same process and collected the same data, supplemented by training on sustainability (GTO step 10). All 29 BGC sites received $2000 a year to defray some costs of study participation—e.g., supplies and snacks used during CHOICE sessions.

### Measures and data collection

PREP was approved by RAND’s Institutional Review Board. Data collectors and technical assistance staff watched for harms of GTO and CHOICE while GTO was active. None were reported.

#### CHOICE fidelity

All sites were rated on three fidelity dimensions—adherence to CHOICE, quality of CHOICE delivery, and dosage [52]. Adherence and quality of CHOICE delivery ratings were made by a pool of eight research data collectors (blind to condition). Rather than calculate reliability by doubling up on observations (which were objectionable to the sites), the entire data collector team rated 16 videotaped sessions developed by the CHOICE trainer, spread over the intervention period. Krippendorff’s α was calculated comparing observers’ ratings for each video to the “master ratings” by the CHOICE trainer.

##### Adherence

Each year, data collectors observed and rated two CHOICE sessions per site (randomly selected) on how closely BGC staff implemented activities as designed (not at all, partially, fully) using a CHOICE fidelity tool [11]. A similar tool was used in the EQUIPS study to rate activities [12] and was found to be reliable (Cohen’s weighted Kappa = 0.92 in year 1; 0.96 in year 2) and valid (showed improvement in the hypothesized direction once GTO was fully implemented in the second year). In each year, a total of 1344 activities were conducted across all 29 sites (a full CHOICE program contains 46 discrete activities). In year 1, we observed and rated 489 activities (36%), distributed across all 29 sites (*n* = 235 for the control group, 254 for the intervention group). In year 2, we observed and rated 515 activities (38%), distributed across all 29 sites (*n* = 255 for the control group, 260 for the intervention group). Ordinal α comparing ratings from each of the eight coders to the master key ranged from .50 to .91, median = .70, acceptable to good by common standards [46].

##### Quality of CHOICE delivery

Motivational interviewing is key to CHOICE delivery. We measured its fidelity with the Motivational Interviewing Treatment Integrity scale (MITI; [53]), the standard measure in clinical trials of motivational interviewing-based interventions [54, 55]. The MITI has five specific behaviors that are counted during the session and five ‘global’ ratings in which the entire session is scored on a scale from 1 = low to 5 = high. The MITI has shown acceptable psychometric characteristics across multiple research settings [56–59], and its scores have correlated with outcomes as expected, suggesting its validity [60–62]. However, because the five global ratings had low inter-rater reliability in this study, we omitted them from analyses and relied on the behavioral data as our operationalization of delivery quality.

The behaviors counted during the session are the number of open- and closed-ended questions, statements that are MI-adherent (e.g., “If it’s ok, I’d like to hear what you thought about that.”) or non-adherent (e.g., “You need to stop using drugs”), and reflections that are simple (e.g., “some of you are ready to make changes”) or complex (e.g., “some of you are hoping that by making changes, things will improve in your lives”). Data collectors administered the MITI at the same sessions they rated adherence. From this count data, we derived four indicators used in the analyses, per the MITI scoring instructions: percent complex reflections (complex reflection/total number of reflections), percent open questions (open questions/total number of questions), reflection to question ratio (total number of reflections/total number of questions), and percent MI adherent (number of MI adherent statements/number of MI adherent + MI non-adherent statements). Across all variables derived from behavioral counts, α for each data collector were in a high range from .88 to .93 [63], median = .90.

##### Dosage

This measure was operationalized as the attendance rates at each site. BGC staff at intervention sites sent RAND the recorded attendance of enrolled youth at each CHOICE session. We calculated attendance (in percent) by dividing the number of youth who completed the baseline survey by the number of youth attending each session, averaged across each session, each year. Control site rates were calculated using attendance data gathered by data collectors during their two visits at each site (averaged together), each year.

#### Youth outcomes

For three substances—cigarettes, alcohol, and marijuana—all youth participants were asked questions about proximal outcomes, which have been shown to predict actual use. Proximal outcome items come from large national surveys, such as monitoring the future [64], and have been used in many different randomized controlled trials with youth [11, 65–67]. All of these measures were used in the previous trials of CHOICE [11, 51]. These measures include intentions to use cigarettes/alcohol/marijuana. Separate single items assessed student intentions to use in the next 6 months (1 = definitely yes to 4 = definitely no) [68]. Resistance self-efficacy (RSE) measured the likelihood one would refuse an offer of cigarettes/alcohol/marijuana in three specific situations (e.g., best friend using, bored at a party, all friends using at a party). Items are rated on a scale from 1 = I would definitely use to 4 = I would definitely not use, and averaged. Higher scores indicated greater RSE (alcohol α = 0.92; marijuana α = 0.96) [69]. Perception of peer use. Youth were asked three separate questions to estimate the percentage of peers in their grade who use cigarettes, alcohol, or marijuana [70]. They were also asked three separate items for whether they think their *best friend uses* these substances [64]. Youth reported on three separate items asking them *how often they spend time around teens* who use cigarettes, alcohol, or marijuana (1 = never to 4 = often) [71]. Positive and negative beliefs were assessed using well-established measures with adolescents [11, 67, 72]. For each substance, youth were asked how much they agreed (1 = strongly agree to 4 = strongly disagree) with various *positive consequences* of using cigarettes (three items; α = 0.78), alcohol (two items; α = 0.81), and marijuana (three items; α = 0.81). Youth were asked the same number of items asking about *negative consequences* for each substance (cigarette α = 0.82; alcohol α = 0.82; marijuana α = 0.83). Because most measures were ordinal and substantially skewed, we dichotomized each variable into lower-risk vs. higher-risk responses for each substance to improve estimation in logistic regression models. We coded intentions: “definitely no” vs. any other; for RSE: “would definitely not use” across all scenarios vs. any other combination; for percentage of peers perceived using in their grade, “0” or “10%” vs. 20% or more; for best friend use, “no” vs. “yes”; for spending time with teens, “never” vs. any other; and for positive and negative consequences, the lowest-risk response across all respective consequences vs. any other combination.

We also asked youth three separate items about their lifetime use and three separate items for their 30-day use of cigarettes/alcohol/marijuana. However, the rates of use were very low. Across the entire sample, the rate of 30-day cigarette use was 1.5% (baseline), 1.4% (3 months), and 1.0% (6 months). For alcohol, it was 3.7%, 4.2%, and 6.7%, respectively. For marijuana, it was 3.3%, 5.9%, and 9.7%, respectively. Because of the low use, the statistical models showed convergence difficulties; therefore, we decided to only analyze the proximal outcomes.

##### Data collection and response rates

In each of the 2 years, the BGC staff in both groups recruited participants for CHOICE from their eligible membership. Staff sent information flyers to parents, approached parents when they were present at the site, and/or held CHOICE information sessions at the site. Parents provided written consent and youth provided assent. RAND (blind to study condition) and BGC staff facilitated paper and pencil youth survey sessions with each site before the launch of CHOICE (Baseline) and at a 3 and 6-month follow-up. We used several methods to boost response rates including make-up sessions, mailed surveys, and phone reminders.

The overall retention rate was 88% at both the 3-month post-test and at the 6-month follow-up. We evaluated differential attrition by condition using discrete-time survival analysis, accounting for clustering within site and BGC, using PROC SURVEYLOGISTIC in SAS. There was a significant effect of assignment to GTO, hazard ratio = 1.09, 95% CI [1.02, 1.16], indicating a greater likelihood of attrition at each follow-up for youth in the GTO group. We also screened for differential attrition by study year, race/ethnicity, gender, age, and lifetime use of different substances at baseline. After applying the Benjamini-Hochberg false discovery rate (FDR) correction, there was a significant difference by gender, with girls more likely to leave the study, HR = 1.06, 95% CI [1.02, 1.10].

### Analyses

#### Overview

We compared intervention and control sites on CHOICE fidelity (adherence, quality of CHOICE delivery, and dosage) and the seven proximal outcomes for cigarettes, alcohol, and marijuana. For each measure, we conducted a mixed model spanning condition and study year, using weighted comparisons to (1) compare intervention and control groups in years 1 and 2 separately; (2) examine year 1-to-year 2 change within intervention and control groups, separately; and (3) assess the interaction term between group and year, to test whether the change from year 1 to 2 differed between groups. All analyses were conducted in SAS v9.4, predominantly with PROC MIXED and PROC GLIMMIX. Effect size estimates were based on spreadsheets provided by Lakens [73, 74]. Confidence intervals for estimated Cohen’s *d* effect sizes were calculated using the SAS macro *Effect_CI* [75].

#### Type I error control

To control the FDR, we adjusted *p* values using the Benjamini-Hochberg procedure [76] such that, across significant findings after adjustment, a proportion of no more than α (.05 herein) reflect type I error. We made this correction within two sets of multiple tests addressing the same conceptual result: the four MI quality indicators and the proximal outcomes analyses for the three substances. Attendance and CHOICE Adherence measures were treated as individual outcomes. We made corrections separately within analyses for year 1, year 2, and the condition by year interaction because these analyses address different conceptual questions. The within-condition change analyses have not been adjusted because they are not part of the study hypotheses and are shown only for illustrative purposes.

#### CHOICE fidelity

We compared control and intervention sites across all three dimensions of fidelity (i.e., CHOICE adherence, MI quality and dosage). For adherence, we fit a mixed-effects, proportional-odds, logistic regression model where the observational unit was one rated CHOICE activity, nested within session, site, BGC, and county. The specific CHOICE activity was included as a rating-level covariate. We report odds ratios and 95% CIs for the treatment effect for the separate year 1 and year 2 analyses, and for the change from year 1 to year 2 by group, and logistic regression coefficient and CI for tests of moderation in the combined years’ analyses. In these models, odds ratios are on an intuitive scale and therefore serve as unstandardized effect sizes. For quality of MI delivery, we tested the site average of each variable in a mixed-effects linear regression model, accounting for nesting within BGC and county. For dosage, we used similar models for respondents’ attendance from the control and intervention groups where the outcome was site-level attendance rate.

#### Youth proximal outcomes

We compared control and intervention youth on seven proximal outcomes across the three substances: cigarettes, alcohol, and marijuana. Because we had no distinct hypotheses about the seven different proximal outcomes, we combined them for each substance into a multivariate mixed model to improve the statistical power of the analysis. For each substance, we fit a mixed-effects logistic regression model where the observational unit was one binary proximal outcome (the lower-risk response) with random intercepts at the levels of youth, site, BGC, and county. Random slope models that we attempted to fit resulted in substantial convergence difficulties. We calculated intraclass correlations (ICCs) for youth intercepts at the level of BGC (the level of random assignment). BGC-level ICCs ranged from .000 to .027 across substances and years, median = .004. We report results in parallel structure as for the adherence data. Again, odds ratios are on an intuitive scale. Youth age, gender, and race/ethnicity (seven binary indicators with multiple selections permitted) were included as covariates in each model, as were baseline measures of all proximal outcomes for all substances.

## Results

### Fidelity

#### Adherence

##### Year 1/year 2 group comparisons

In year 1, there were no significant difference between groups in the probabilities of the three protocol adherence ratings (“not at all,” “partially,” “fully”) (see Table 2 for details). Table 2 includes by-condition descriptive and test statistics and an unstandardized odds ratio as an effect estimate for year 1 (leftmost) and year 2 (center). In year 2, intervention sites scored significantly higher on the 3-point adherence scale than control sites. For the intervention group, 2% of activities in year 2 were rated as not at all adherent, 11% as partially adherent, and 87% as fully adherent, similar to the 90% fully adherent ratings in the cluster-randomized trial of CHOICE [11]. For the control group, the comparable numbers were 12%, 22%, and 66%, respectively.

**Table 2:** Adherence to CHOICE Activities in years 1 and 2

	Year 1						Year 2						Change from Year 1 to Year 2, Odds Ratio (95% CI), *t*(909)		
How well was the CHOICE activity completed?	Control		Intervention		*t* (909)	Odds Ratio (95% CI)	Control		Intervention		*t* (909)	Odds Ratio (95% CI)	Control	Intervention	Difference of Differences
	Freq	%	Freq	%			Freq	%	Freq	%					
Fully	171	68	162	65	-0.27	0.92 (0.49, 1.73)	171	66	206	87	4.21	4.40 (2.20, 8.77)*	0.85 (0.56, 1.27) -0.80	4.07 (2.44, 6.77)* 5.39	Logistic *b* = 1.57 (0.91, 2.22)* 4.69
Partially	63	25	70	28			58	22	27	11					
Not at all	19	8	17	7			32	12	4	2					
Number of activity observations	253		249				261		237						

*Note: N* = 1000; *k*(sites) = 29, *k*(BGCs) = 15

Differences in changes in adherence ratings where noted with the following asterisk: *p < .05

##### Year 1 to year 2 within group change

As shown in the right-hand portion of Table 2, comparing years 1 and 2, the intervention group had significantly higher ratings of adherence to CHOICE activities in year 2 than in year 1. The control group showed no significant change.

##### Year 1 to year 2 interactions

The difference in change between the two groups was significant (rightmost column of Table 2). As noted, in year 2, the control group ratings were essentially unchanged from year 1. However, those in the intervention group increased their adherence to the protocol activities by almost four-fold in year 2 (defined as going from “not at all,” to “partially,” or “fully”; going from “partially” to “fully”).

#### Quality of CHOICE delivery

##### Year 1/year 2 group comparisons

Across all four MI quality variables, mixed-effects regression models showed no group differences in year 1, *p*s > .20 (Table 3). The median estimated Cohen’s *d* was − 0.18. In year 2, however, the intervention group had significantly higher MI quality ratings than the control group for two of the four quality variables. After FDR correction, these differences significantly favored the intervention group for the derived reflection question ratio and for percent MI adherent. The median *d* across the four ratings was 0.74. According to thresholds for proficiency established by the MITI [53] (reflection to question ratio = 1.0; percent open questions = 50%; percent complex reflections = 40%; and percent MI adherent = 90%), in year 2, the intervention sites were either near or over these thresholds on three of the four measures (.88, 64%, 32%, 95%, respectively). Control sites were over the threshold for percent open questions only (.81, 59%, 26%, 81%, respectively). Thus, results indicate that the intervention sites were delivering CHOICE with higher MI quality than the control sties in year 2. Further, MI adherent scores for the intervention group (year 1: 94%, year 2: 95%) were very similar to what was reported in the cluster-randomized trial of CHOICE (93%) [11].

**Table 3:** Motivational interviewing quality and CHOICE dosage in years 1 and 2

	Year 1^1^				Year 2^1^				Change from year 1 to year 2 ^2^, Odds ratio (95% CI)		
Quality of delivery	*M* (SD)		*t* (54)	*d* (95% CI)	*M* (SD)		*t* (54)	*d* (95% CI)	*M*_diff_ (95% CI), *t* (39), Cohen’s *d* (95% CI)		*F* (1, 54), partial ω^2^
	Control (*N* = 15)	Intervention (*N* = 14)			Control (*N* = 15)	Intervention (*N* = 14)			Control	Intervention	Difference of differences
Percent complex reflections	32 (14)	28 (16)	− 0.78	− 0.29 (− 1.02, 0.44)	26 (15)	32 (9)	1.19	0.44 (− 0.30, 1.18)	− 6 (− 13, 1), − 1.62, − 0.42 (− 1.14, 0.31)	4 (− 3, 12), 1.12, 0.30 (− 0.45, 1.04)	3.73, .046
Percent open questions	59 (14)	58 (13)	− 0.16	− 0.06 (− 0.79, 0.67)	59 (15)	64 (17)	0.75	0.28 (− 0.46, 1.01)	1 (− 9, 10), 0.16, 0.04 (− 0.68, 0.76)	6 ( 4, 17), 1.23, 0.33–0.42, 1.07)	0.60, −.007
Reflection question ratio	.67 (.19)	.66 (.32)	− 0.16	− 0.06 (− 0.79, 0.67)	.56 (.24)	.88 (.29)	3.07*	1.14 (0.34, 1.92)	− 0.11(− 0.30, 0.08), − 1.18, − 0.30 (− 1.02, 0.42)	0.22 (0.03, 0.42), 2.33^, 0.62 (− 0.14, 1.38)	6.24, 086
Percent MI adherent	88 (16)	94 (8)	1.27	0.47 (− 0.27, 1.21)	81 (21)	95 (7)	2.80*	1.04 (0.25, 1.81)	− 8 (− 18, 2), − 1.59, − 0.41 (− 1.13, 0.32)	1 (− 9, 11), 0.23, 0.06 (− 0.68, 0.80)	1.62, .011
Dosage	Control (*N* = 11)	Intervention (*N* = 14)	*t* (50)	*d* (95% CI)	Control (*N* = 15)	Intervention (*N* = 14)	*t* (50)	*d* (95% CI)	*M*_diff_ (95% CI), *t* (37), *d* (95% CI)		*F* (1, 50) partial ω^2^
Percent attendance across sessions, *M* (SD)	76 (14)	79 (13)	0.69	0.28 (− 0.52, 1.07)	69 (13)	75 (9)	1.38	0.51 (−0.23, 1.25)	− 7 (− 15, 2), − 1.51, − 0.42 (− 1.19, 0.36)	−4 (− 12, 5), − 0.89, − 0.24 (− 0.98, 0.51)	0.25, −.015

*Note: N* = 54; *k*(sites) = 29, *k*(BGCs) = 15

^1^Tests comparing performance ratings between the intervention and control groups within year

^2^Tests comparing performance ratings between years 1 and 2 within and between groups

*Performance ratings were significantly higher for the intervention group after false discovery rate adjustment, *p* < .05

^^^Significant differences in change from year 1 to year 2 within group

##### Year 1 to year 2 within group change

Only the reflection question ratio improved from year 1 to 2 in the intervention group, *p* = .023. None of the measures showed significant year-to-year improvement in the control group.

##### Year 1 to year 2 interactions

None of the measures showed significantly different change over time between conditions after FDR. The median estimated partial ω^2^ for the interaction term was .028.

#### Dosage (attendance)

The control and intervention groups did not differ in their attendance in years 1 or 2 or in change between years 1 and 2, *p*s > .05.

### Youth outcomes

#### Year 1/year 2 group comparisons

Table 4 has the proportions of youth endorsing a lower risk response for each proximal outcome by year, study group, and substance. Table 5 includes by-condition descriptive and test statistics and an unstandardized odds ratio as an effect estimate for year 1 (leftmost) and year 2 (center). In year 1, the mixed-effects logistic regression model found no significant difference between groups in the probabilities of endorsing the lower-risk response in the dichotomized proximal outcomes. The median odds ratio across the six comparisons was 0.94; based on absolute value of the logistic coefficient, the median effect size was OR = 1.14. In year 2, there were also no significant differences for the alcohol, cigarette, or marijuana outcomes after FDR correction. The median odds ratio was 1.12; median effect size OR = 1.23.

**Table 4:** Proportion of lower risk response for proximal outcomes by year, study group, and substance

		Year 1						Year 2					
		Control			Intervention			Control			Intervention		
Substances	Proximal outcomes (items)	BL	3 months	6 months	BL	3 months	6 months	BL	3 months	6 months	BL	3 months	6 months
Cigarettes	Positive consequences^1^ (3)	.59	.45	.43	.53	.49	.49	.55	.50	.55	.62	.47	.51
	Negative consequences^2^ (3)	.30	.36	.41	.30	.39	.27	.27	.38	.42	.28	.38	.42
	Resistance self-efficacy^3^ (3)	.80	.81	.81	.79	.81	.75	.82	.83	.80	.84	.84	.77
	Perception of peer use^4^ (1)	.71	.71	.74	.72	.70	.70	.80	.72	.72	.79	.66	.77
	With using friends^5^ (1)	.76	.76	.80	.75	.78	.74	.79	.80	.80	.81	.75	.80
	Best friend use^6^ (1)	.94	.96	.95	.91	.96	.95	.95	.95	.94	.91	.91	.94
	Intentions to use^7^ (1)	.92	.93	.92	.91	.91	.91	.91	.93	.95	.80	.87	.93
Alcohol	Positive consequences (2)	.64	.53	.52	.56	.54	.46	.62	.56	.56	.64	.57	.58
	Negative consequences (2)	.59	.72	.67	.59	.61	.57	.54	.60	.61	.56	.65	.70
	Resistance self-efficacy (3)	.80	.74	78	.77	.78	.68	.80	.77	.78	.75	.79	.74
	Perception of peer use (1)	.72	.63	.66	.69	.64	.63	.78	.70	.65	.74	.65	.64
	With using friends (1)	.73	.76	.80	.70	.71	.64	.78	.73	.78	.79	.78	.76
	Best friend use (1)	.90	.89	.91	.88	.85	.87	.96	.90	.89	.85	.89	.89
	Intentions to use (1)	.82	.83	.85	.82	.83	.77	.86	.85	.88	.89	.90	.84
Marijuana	Positive consequences (3)	.56	.49	.46	.52	.51	.47	.59	.59	.51	.62	.56	.48
	Negative consequences (3)	.79	.82	.78	.76	.79	.71	.75	.72	.74	.73	.74	.78
	Resistance self-efficacy (3)	.86	.86	.88	.84	.87	.74	.89	.85	.86	.86	.84	.80
	Perception of peer use (1)	.68	.65	.67	.67	.61	.62	.74	.65	.66	.75	.58	.68
	With using friends (1)	.77	.76	.77	.68	.72	.65	.79	.76	.72	.75	.71	.71
	Best friend use (1)	.87	.93	.89	.88	.87	.84	.97	.91	.85	.89	.86	.86
	Intentions to use (1)	.89	.90	.91	.90	.88	.82	.93	.90	.89	.90	.87	.86

*BL* = baseline; *3 months* = 3-month follow-up after program; *6 months* = 6-month follow-up after program

^1^Positive consequences. Lower risk = “strongly disagree” across all consequences vs. any other combination

^2^Negative consequences. Lower risk = “strongly agree” across all consequences vs. any other combination

^3^Resistance self-efficacy. Lower risk = “would definitely not use” across all situations

^4^Perception of peer use. % of peers in their grade who they believe use; lower risk = “0” or “10%” vs. 20% or more

^5^With using friends. Lower risk = “never”

^6^Best friend use. Lower risk = “no”

^7^Intentions to use. Lower risk = “definitely no”

**Table 5:** Youth proximal outcomes in years 1 and 2

Substance	Intervention effect in year 1		Intervention effect in year 2		Change from year 1 to year 2 within condition^2^		
	*t*	Odds ratio (95% CI)	*t*	Odds ratio (95% CI)	Odds ratio (95% CI), *t*		Logistic *b* (95% CI), *t*
					Control	Intervention	Difference of differences
Cigarettes							
3 months	1.10	1.20 (0.87, 1.65)	− 1.39	0.76 (0.51, 1.12)	1.04 (0.74, 1.47), 0.23	0.66 (0.46, 0.95), − 2.22^	− 0.46 (− 0.96, 0.05), − 1.77
6 months	− 0.49	0.92 (0.68, 1.27)	− 0.92	0.83 (0.56, 1.23)	1.10 (0.78, 1.55), 0.53	0.98 (0.68, 1.42), − 0.08	− 0.11 (−.0.62, .40), − 0.42
Alcohol							
3 months	− 0.44	0.93 (0.68, 1.27)	1.96	1.48 (1.00, 2.19)	0.79 (0.56, 1.11), − 1.35	1.25 (0.87, 1.80), 1.21	0.46 (− 0.04, 0.96), 1.80
6 months	− 1.86	0.75 (0.55, 1.02)	1.11	1.25 (0.84, 1.85)	0.79 (0.56, 1.12), − 1.33	1.33 (0.92, 1.91), 1.53	0.51 (0.02, 1.01), 2.02
Marijuana							
3 months	− 0.29	0.95 (0.68, 1.33)	− 0.29	0.95 (0.68, 1.33)	0.71 (0.49, 1.03), − 1.81	0.78 (0.53, 1.16), − 1.22	0.10 (− 0.44, 0.64), 0.35
6 months	0.52	1.12 (0.73, 1.70)	0.52	1.12 (0.73, 1.70)	0.66 (0.46, 0.96), − 2.20^	0.99 (0.67, 1.47), − 0.04	0.40 (− 0.13, 0.93), 1.47

*Note:* Minimum response *N* = 11,001; *k*(youth) > = 506, *k*(sites) = 29, *k*(BGCs) = 15. Minimum degrees of freedom for *t* statistics = 10,409

^#^A negative number indicates a decline in the rate of endorsing a lower-risk option; a positive number indicates an increase in the rate of endorsing a lower-risk option

^1^Tests comparing lower-risk response rates between the intervention and control groups within year

^2^Tests comparing lower-risk response rates between years 1 and 2 within and between groups

*p < .05, after false discovery rate adjustment within column

^Significant differences in change from year 1 to year 2 within group

#### Year 1 to year 2 within group change

As shown in the right-hand portion of Table 5, between years 1 and 2, the control group showed fewer youth endorsing a lower risk response for the 6-month follow-up marijuana proximal outcomes in year 2 than in year 1. The intervention group showed fewer youth endorsing a lower risk response for the 3-month cigarette proximal outcomes in year 2 than in year 1.

#### Year 1 to year 2 interactions

As detailed in the rightmost column of Table 5, there were no significant differences between conditions in year-to-year change.

### Sensitivity analyses

We conducted sensitivity analyses to determine whether distorted or arbitrary response sets from youth or inconsistent coding of CHOICE adherence could have resulted in different outcomes. First, to address the possibility of distorted or arbitrary response sets, we asked youth about their use of a non-existent drug called “derbisol.” Across all surveys and waves, 12 participants reported using derbisol at least once. Youth outcome analyses excluding these participants resulted in no differences in interpretation of results. Second, the range of reliability of the coders for CHOICE adherence was substantial. We repeated the CHOICE adherence analyses excluding ratings contributed by the two judges with coding reliability < .65. This analysis likewise resulted in no differences in interpretation of results.

## Discussion

The PREP study assessed GTO’s impact on an evidence-based program’s fidelity and youth proximal outcomes over 2 years. In year 1, intervention and control sites were similar on adherence. Both groups carried out about two-thirds of CHOICE activities in full and about one quarter of CHOICE activities in part, far below the original CHOICE trial. In year 2, as hypothesized, the intervention sites significantly improved adherence, implementing CHOICE activities fully 87% of the time (similar to sites implementing CHOICE in its original trial), while control sites were unchanged.

There was a similar pattern of results between years 1 and 2 for the MI delivery quality. None of the four MI variables were significantly different between the two groups in year 1. However in year 2, as hypothesized, the intervention sites had higher ratings on reflection to question ratio and percent MI adherent and had greater improvement on reflection question ratio from year 1 to 2 compared to the control sites. The intervention sites achieved the same percent MI adherence ratings as sites in the original CHOICE trial. Dosage (i.e., attendance) was not different between the groups in either year. The improvement in fidelity with implementation support documented here is similar to other studies testing implementation support models among alcohol and drug prevention evidence-based programs [77–79].

The similarity of the two groups’ fidelity scores in the first year could be because control sites received some GTO-like support simply by being in the study and because the CHOICE materials and training provide some guidance. We concluded this in part because we provided CHOICE training to three other youth-serving organizations in Los Angeles, at no cost, and then after having no contact with them for 6 months, asked them whether they ran any programming. None ran any CHOICE programming, suggesting that the control group’s act of participating in the study might induce some level of program implementation that would not otherwise occur. In the second year, with the addition of GTO’s quality improvement activities, in which plans were developed to specifically improve identified areas of weakness, the intervention group’s adherence and quality delivery ratings were much higher, as hypothesized. Thus, we conclude that working through the evaluation and quality improvement steps of GTO is important to achieving intended outcomes. This often takes time and requires more than 1 cycle of a program, since the benefits of quality improvement are not realized until they are applied to a subsequent program implementation.

Compared to the implementation findings, the youth proximal outcome results did not change much from baseline to 6 months in either year and thus did not show any clear differences between groups, contrary to our hypothesis. Direct outcome comparisons to the earlier CHOICE trial are not possible because that study involved youth with higher rates of substance use and the PREP youth tended to endorse responses to the proximal outcomes that were somewhat lower risk. Thus, one reason the proximal outcomes did not show larger change in our study could be due to a lack of variability in these outcomes—i.e., there was generally high endorsement of low risk responses in our sample. Similar to other studies of implementation support [78], improvements in implementation fidelity do not always translate into better program outcomes.

PREP’s findings are similar to the implementation results of EQUIPS. Like PREP, EQUIPS showed that sites using GTO had better fidelity results (i.e., adherence and delivery quality ratings) in year 2. In the EQUIPS study, we concluded that “in typical community-based settings, manuals and training common to structured evidence-based programs may be sufficient to yield….moderate levels of fidelity, but that more systematic implementation support is needed to achieve high levels of performance and fidelity [12, pg. 14].” The findings of the PREP study, using a different evidence-based program and measures of fidelity, appear to bolster that conclusion. However, the PREP study goes further, suggesting that GTO can help community-based practitioners carry out with proficiency a more complicated set of skills required by motivational interviewing. This is important as adolescents tend to be more satisfied with interventions that use motivational interviewing and facilitators that use these skills can be more effective in preventing or reducing substance use [80]. Thus, supports like GTO could significantly improve the generally poor implementation of evidence-based programs among youth-serving organizations across a range of prevention programs.

Both EQUIPS and PREP were carried out in Boys and Girls Clubs and are thus generalizable to low-resourced, community-based settings. It is possible that organizations with greater resources and more staff could achieve even better results with GTO. These results were achieved with a modest amount of training and technical assistance time (about 26 h over the 2-year intervention period), which is similar to what government grant programs now offer [81]. A cost analysis is underway from the PREP study that will provide more information about GTO’s return on investment.

There are some limitations that should be noted. First, it was difficult to evaluate GTO’s effects on substance use and proximal outcomes. The sample of youth in this study, ages 10 to 15, had very low base rates of substance use and high rates of endorsing prosocial responses on Proximal outcomes (more than in the original CHOICE trial). More youth in the nearby public school system (of the same age) were Latinx (74% vs. 64%) and fewer were African-American (9% vs. 17%) than the youth in our sample. A great deal of research has shown that African American youth tend to report less substance use than other races/ethnicities [82–88], so one reason for our lower rates of substance use and high positive proximal outcomes may be because we had more African American youth participate in the study. Alternatively, Boys and Girls Clubs are focused on helping youth make healthy choices across a variety of behaviors; and thus, these youth may have already been exposed to more preventive programming than youth in the CHOICE trials and the nearby public schools system. Second, sites did not have the full experience in doing a needs assessment or searching for and choosing an evidence-based program (GTO steps 1 and 3, respectively). Instead, club leaders agreed to carry out a single evidence-based program (i.e., CHOICE) prior to the study. Use of a single evidence-based program better isolates the effects of GTO between study groups. For all other GTO steps, each site individually carried out the related practices. Given the similarity among many universal alcohol and drug prevention programs, we believe GTO received a strong test in PREP. Understanding the impact of program choice on implementation and outcomes is an important topic for future studies. Third, we were only able to study two program cycles of CHOICE. Future studies should examine whether continued cycles using GTO confers even greater benefits to programs than we were able to document here. Fourth, staff were aware of the study group they were in because each staff person learned about the study during the consent process. It is possible that those in the intervention group were additionally motivated to conduct CHOICE with fidelity because of their knowledge of their group membership. Finally, smaller effects may have gone undetected given the number of sites was 29; substantial for an RCT, but modest for testing site-level outcomes. Future rigorous studies are needed in which the impact of implementation support is assessed on the large scale used in federally or state-funded initiatives [89–92].

## Conclusions

Community-based practitioners using GTO to carry out an evidence-based alcohol and drug prevention program demonstrated better fidelity than practitioners not using GTO after 2 years. Findings replicate the implementation results of a previous GTO study using the same design, but with a different, more challenging evidence-based program, content domain, and fidelity measures. Improved implementation did not translate into better individual youth outcomes, in large part because the youth had very low drug and alcohol use and generally positive proximal outcomes from the start. However, given typically poor evidence-based program implementation nationwide, these findings are significant as they highlight that GTO can improve the implementation of programs that use complex delivery methods and operate across multiple domains.

This project is registered at ClinicalTrials.gov with number NCT02135991. The trial was first registered on May 12, 2014.

## Abbreviations

BGC(s): Boys and Girls Club(s)
EQUIPS: Enhancing Quality Interventions Promoting Healthy Sexuality
GTO: Getting To Outcomes
IRR: Inter-rater reliability
PREP: Preparing to Run Effective Prevention
RCT: Randomized controlled trial
